# Characteristics of Pivotal Trials and FDA Review of Innovative Devices

**DOI:** 10.1371/journal.pone.0117235

**Published:** 2015-02-04

**Authors:** Joshua P. Rising, Ben Moscovitch

**Affiliations:** The Pew Charitable Trusts, Washington, District of Columbia, United States of America; The University of Auckland, NEW ZEALAND

## Abstract

When patients lack sufficient treatment options for serious medical conditions, they rely on the prompt approval and development of new therapeutic alternatives, such as medical devices. Understanding the development of innovative medical devices, including the characteristics of premarket clinical trials and length of Food and Drug Administration (FDA) review, can help identify ways to expedite patient access to novel technologies and inform recent efforts by FDA to more quickly get these products to patients and physicians. We analyzed publicly available information on clinical trials and premarket FDA review for innovative medical devices that fill an unmet medical need. In this first-of-its-kind study focusing on these products, we extracted data on the length of the pivotal trials, primary study endpoint and FDA review; number of patients enrolled in trials; and in what country the device was available first. We identified 27 approved priority review devices from January 2006 through August 2013. The median duration of pivotal clinical trials was 3 years, ranging from 3 months to approximately 7 years. Trials had a median primary outcome measure evaluation time of one year and a median enrollment of 297 patients. The median FDA review time was 1 year and 3 months. Most priority review devices were available abroad before they were approved in the United States. Our study indicates that addressing the length of clinical studies—and contributing factors, such as primary outcome measures and enrollment—could expedite patient access to innovative medical devices. FDA, manufacturers, Congress and other stakeholders should identify the contributing factors to the length of clinical development, and implement appropriate reforms to address those issues.

## Introduction

A recent proposal from the Food and Drug Administration (FDA) to speed patient access to innovative medical devices that fill unmet needs would shift some of the evidence typically collected during product development to after the technology comes to market [1, 2]. Currently, most medical devices are reviewed under one of two pathways. High-risk devices (also known as Class III products) are generally assessed through the premarket approval (PMA) process, under which manufacturers must conduct at least one clinical study and submit their data for FDA review. Low- and medium-risk products (class I and II products) that require FDA review are usually evaluated on whether they are substantially equivalent to already marketed devices; these products only occasionally require clinical trial data. FDA aims to complete review of PMA applications within 180 days (320 days if the evaluation requires input from a federal advisory committee) and review of applications which seek to demonstrate substantial equivalence within 90 days of receiving an application [3]. FDA also reviews a very small number of low- and medium-risk products that are not substantially equivalent to previously marketed devices through a *de novo* process and evaluates the safety of devices that treat or diagnose less than 4,000 patients annually via its humanitarian device exemption (HDE) program.

FDA’s regulation of medical devices differs significantly from the process used in Europe, where manufacturers of high-risk products use for-profit organizations—known as Notified Bodies—to evaluate that the product is safe and functions as described, but not that the technology is effective or provides a clinical benefit to the patient [4]. Notified Bodies often do not provide information to the public on the data reviewed, or even when they approve a product—known as granting a CE Mark. Once the product obtains a CE Mark from a Notified Body, it may be marketed in the European Union. The differences between EU and US device premarket review have led to conflicting interpretations of the European experience, with some stating that the European approach is better since many devices get approved first there [5] and others pointing to recent safety problems with devices in Europe as evidence that European regulation of devices should be strengthened [6, 7].

In an effort to help innovative products reach patients more quickly, the FDA proposed a new expedited access PMA (EAP) program, under which the agency could defer some data collection for a product until after the technology is marketed for use in patient care [8]. Under this new program—designed for devices that fill a serious, unmet medical need—FDA could accept smaller trials, shorter follow-up times or the use of surrogate endpoints with the stipulation that manufacturers will ultimately provide the agency with the type and amount of evidence typically required to gain approval. For example, manufacturers could hypothetically demonstrate before approval that a novel cardiac implant safely improves blood pressure in treatment-resistant patients; FDA could require the manufacturer to collect postmarket data on the rate of heart attacks and mortality and provide it to the agency to meet the FDA’s safety and efficacy standards. For these products, FDA would work with the manufacturer to develop a data development plan to outline the information needs at different points in the device lifecycle, provide additional feedback to the manufacturer during development, and facilitate development in several other ways. In parallel, FDA released another policy to apply some of these same principles of shifting data collection to after approval to a much broader subset of devices—not necessarily just those deemed innovative [1]. Through these new policies, clinicians could have new tools to treat, cure and diagnose their patients more quickly. Providers and patients, though, must also have assurances that the benefits outweigh the risks of new devices used; postmarket data collection would be essential to meet that need.

To better understand the potential of this proposal to get certain devices to market more quickly, we conducted the first research—to our knowledge—to provide data on the length of clinical development time and FDA review for innovative devices. Previous studies have found that clinical development for devices in general—regardless of whether they are variations of existing technology or novel products—can take many years [9], and that FDA review of PMAs lasted between one and two years on average [10], though that time has recently decreased [11].

To better understand the development process and regulatory evaluations for the most novel products, we studied medical technologies that qualified for FDA’s priority review program, which is designed to expedite patient access to new innovative devices by requiring the agency to evaluate these applications ahead of other products. Examples of devices approved through priority review include a transcatheter artificial heart valve for patients deemed inoperable for open aortic valve replacement, an ablation system that non-invasively erodes metastatic cancer, and technology that delivers radiofrequency energy to the lungs of patients whose severe persistent asthma is not well controlled with other treatments.

To obtain a priority review designation—which was formerly referred to as expedited review—a device must treat a serious, unmet medical need, such as a life-threatening illness for which inadequate therapy exists [12]. FDA has proposed applying the same criteria that it currently uses to make priority review designations for its new EAP pathway to accelerate the delivery of novel devices to patients.

## Methodology

We limited our research to devices granted premarket approval (PMA), an FDA review designation for the highest risk devices. We did not include products cleared through demonstrating substantial equivalence since these products are, by definition, similar to other devices and therefore less likely to represent significant innovations.

We focused our research on devices with priority review status because the criteria for this designation are the same as FDA outlines in its new policy. We could only obtain data on approved products, as information on unapproved devices is not publicly available. FDA was able to provide basic aggregated information on products that were rejected by the agency or withdrawn from review during our study timeframe. The applicable aggregate data from FDA is included in this paper as it was received from the agency. We further restricted our analysis to device reviews completed from January 2006 through August 2013 because data prior to that time were often incomplete.

We examined clinical studies considered pivotal to FDA review. Each device application typically has one study that gathers the final data on safety and effectiveness needed for FDA review [13]. One device had two pivotal trials; for the purposes of this study, we combined the data from these two trials.

We sought to understand the length of the pivotal trial, the length of time between the end of data collection and the FDA receipt date of the application, the FDA review time and the total time from the start of the trial to FDA approval. Additionally, we sought information on the number of enrollees in pivotal trials and the length of the primary endpoints. Finally, we examined the availability of the product abroad at the time the application was submitted to FDA.

We used FDA’s PMA database to identify priority review devices and provide basic information [14]. We then reviewed each product’s online Summary of Safety and Effectiveness Data (SSED), which includes information on the reason that FDA granted priority review and pivotal trial data, as has been done in similar studies examining device development [15]. In some cases, the SSED lacked complete data. In these cases, we supplemented the data with information in documents from FDA advisory committee meetings [16] and the National Institutes of Health’s clinical trials database [17].

To ensure the accuracy of data entry, each of us extracted information on half the devices; then, the other would confirm data on each entry. We jointly resolved discrepancies.

We calculated the mean and median of the following: length of the pivotal trial (as defined from the start date of a study to the date of database lock or primary endpoint completion if we did not have the lock date); length of FDA review from the date of submission to approval (which includes time when the agency is not evaluating the application and is awaiting additional information from the sponsor); time from the initiation of pivotal trials to FDA approval; and length of primary outcome measures. For dates with only the month and year listed, we used the first of the month. We converted days to months, using 30 days as equal to one month for our calculations.

The calculation of the length of FDA review includes time when FDA is not evaluating the application as the agency awaits additional information from the product sponsor. We could not calculate only the time when FDA is reviewing the application because that information is not publicly available for each application; the only available information is the aggregate length of FDA review including the time that manufacturers prepare responses to agency questions.

In calculating the primary measure evaluation time, we omitted devices that did not require patient follow-up.

For each device we also calculated the time from the end of a trial to FDA’s receipt of an application. We sought to understand how often FDA receives an application before the end of a trial, which can occur, for example, if the device sponsor submits an application before locking the database and then updates the data during FDA review. The data used in this study did not indicate, for each device, why device sponsors submitted applications before the end of the database lock. Due to this phenomenon, we did not calculate the mean and median for these data.

Last, we calculated the mean and median trial enrollment, including patients that dropped out or were lost to follow up. In some cases, the enrollment figure represents the number of procedures. This occurred, for example, with data on a hip implant where the number of hips replaced—not number of patients—represent the primary data point in the trial.

**Fig 1:**
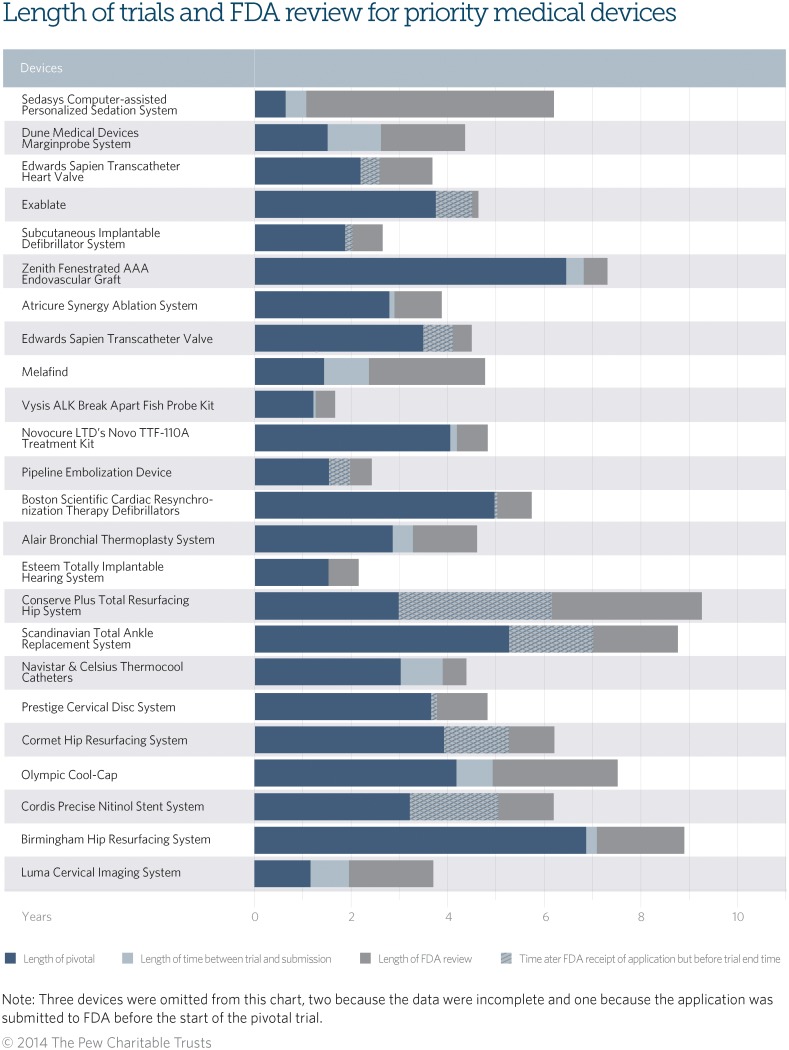
Graph—Characteristics of Pivotal Trials and FDA Review of Innovative Devices: This graph depicts the lengths of clinical trials, time between the trial and FDA review, and FDA review.

Table 1 illustrates some of the clinical trial and FDA review characteristics of each product. Fig. 1 depicts the different phases in development for each device. The dark blue bar represents the length of the pivotal clinical trial and the grey section corresponds to the FDA review time. The light blue or checkered sections represent the time between the end of the trial and submission to FDA. Where this light blue box is checkered, this represents the time when the pivotal trial had not been completed but the application was already submitted to FDA.

**Table 1:** Characteristics of Pivotal Trials and FDA Review of Innovative Devices.

Device (Approved Year)	Description	Length of pivotal trial (days)	Length of FDA review (days)	Length pivotal start to approval (days)	Length of primary endpoint completion (N/A occurs for diagnostics and other devices with no follow-up) (days)	Enrollment	Already available abroad (N/A occurs because the approval is for a device already available to U.S. patients, either for a different indication or as a supplement)	Data sources [14] [16, 17]
Sedasys Computer-assisted Personalized Sedation System (2013)	Computer-assisted personalized sedation system that administers propofol for minimal-to-moderate sedation; monitors and alarms for physiological vital signs of sedation and limits the depth of sedation.	232	1865	2250	N/A—no follow-up	1000	Yes	SSED
Dune Medical Devices Marginprobe System (2012)	Radiofrequency spectroscopy probe system that measures the properties of breast lumpectomy tissue and characterizes it as cancerous or normal.	546	633	1578	60	664	Yes	SSED; clinicaltrials.gov
Edwards Sapien Transcatheter Heart Valve (2012)	Transcatheter heart valve for patients at high risk of mortality from surgical aortic valve replacement; expanded indication for transapical delivery, among other changes.	934	536	1328	360	1057	N/A—available for a different indication	SSED; advisory committee documents
Exablate (2012)	Magnetic resonance ultrasound surgery device used to non-invasively target and ablate tissues; indicated for patients suffering from bone pain due to metastatic disease and for whom standard radiation therapy fails or is not an option.	1645	318	1692	90	139	N/A—available for a different indication	SSED; clinicaltrials.gov
Subcutaneous Implantable Defibrillator System (2012)	Subcutaneous electrode to sense and detect ventricular arrhythmias and deliver defibrillation shocks.	748	280	975	180	330	Yes	SSED
Zenith Fenestrated AAA Endovascular Graft (2012)	Endovascular stent graft used instead of open surgery in patients with abdominal aortic or aorto-iliac aneurysms.	2342	180	2650	180	42	N/A—supplement	SSED
Atricure Synergy Ablation System (2011)	Heart tissue ablation system indicated for patients with persistent or longstanding atrial fibrillation who are undergoing open concomitant coronary artery bypass grafting and/or valve replacement or repair.	1013	356	1405	180	55	N/A—available for a different indication	SSED
Edwards Sapien Transcatheter Valve (2011)	Heart valve delivered via a transcatheter approach meant as an alternative for patients deemed inoperable for open aortic valve replacement.	1492	366	1636	360	358	Yes	SSED
Melafind (2011)	Optical imaging and analysis device to detect melanoma among atypical skin lesions.	523	875	1735	N/A—diagnostic	1831	Yes	SSED
Vysis ALK Break Apart Fish Probe Kit (2011)	Laboratory test that detects the presence of chromosomal rearrangements of the ALK gene in non-small cell lung cancer tissue samples to aid in identifying patients eligible for treatment with the cancer drug crizotinib.	440	147	602	N/A—diagnostic	136	No	SSED
Novocure LTD’s Novo TTF-110A Treatment Kit (2011)	Portable device that generates alternating electrical fields to treat patients with histologically confirmed glioblastoma multiforme; intended as an alternative to standard medical therapy when surgical and radiation options have been exhausted.	1470	235	1753	180	237	Yes	SSED
Pipeline Embolization Device (2011)	Permanent implant combined with a guidewire-based delivery system placed within the internal carotid artery; intended for adults with wide-necked intracranial aneurysms as an alternative to embolic coils or a liquids.	718	323	884	180	111	Yes	SSED
Boston Scientific Cardiac Resynchronization Therapy Defibrillators (2010)	A ventricular antitachycardia pacing and defibrillation system indicated for patients with mild heart failure.	1835	279	2094	1020	1820	N/A—supplement	SSED
Implantable Miniature Telescope (2010)	Implanted intraocular telescope systems to improve vision in patients at least 75 years old with stable, severe, or profound vision impairment caused by end-stage age-related macular degeneration.	Not included because we lacked an accurate trial end date.	Not include in sample since FDA issued a not approv-able letter.	Not included in sample because we lacked an accurate trial end date and date for the commen-cement of FDA review after the not approv-able letter.	360	217	Yes	SSED; FDA advisory committee documents
Alair Bronchial Thermoplasty System (2010)	Single-use disposable device designed to provide controlled delivery of radiofrequency energy directly to the lungs; indicated for treatment of severe persistent asthma in adults whose asthma is not well controlled with inhaled corticosteroids and long acting beta agonists.	1035	483	1669	360	297	No	SSED; clinicaltrials.gov
Esteem Totally Implantable Hearing System (2010)	First totally implantable hearing system to treat moderate to severe hearing loss in adults caused by defective inner ear function; provides an alternative to non-implantable and partially implantable hearing aid technology.	556	225	785	300	60	Yes	SSED
Conserve Plus Total Resurfacing Hip System (2009)	Total hip system with resurfacing femoral component and a metal-on-metal articulation; intended for use in resurfacing hip arthroplasty for reduction or relief of pain and/or improved hip function in patients with degenerative joint diseases.	2274	2226	3353	720	1366	Yes	SSED
Scandinavian Total Ankle Replacement System (2009)	An artificial ankle joint replacement, indicated for use as a non-cemented implant to replace a painful arthritic ankle joint due to osteoarthritis, post-traumatic arthritis or rheumatoid arthritis.	2540	1267	3176	720	224	Yes	SSED
Navistar & Celsius Thermocool Catheters (2009)	An ablation catheter threaded through the femoral vein to ablate abnormal heart tissue that causes atrial flutter despite drug therapy.	1095	177	1589	360	167	N/A—supplement	SSED; clinicaltrials.gov
Freestyle Navigator Continuous Glucose Monitor (2008)	Glucose sensor that reports glucose values continuously for up to 120 hours; detects trends and patterns in glucose levels in adults with diabetes.	99	1008	917	40	137	No	SSED
Prestige Cervical Disc System (2007)	Two-piece metal device attached to neck bones with bone screws to replace a diseased cervical discs from C3–C7 following removal of the disc for conditions resulting from a diseased or bulging disc.	1369	423	1749	720	541	Yes	SSED
Cormet Hip Resurfacing System (2007)	Metal-on-metal hip resurfacing system meant for reduction or relief of pain and/or improved hip function in patients with increased activity level who may not be suitable for traditional total hip arthroplasty.	1896	825	2238	720	1154	Yes	SSED; FDA advisory committee documents
Guardian Real-Time and Paradigm Real-Time System (Pediatric) (2007)	Glucose sensors that report glucose values every 5 minutes for up to 72 hours; detects trends and patterns in glucose levels in adolescents with diabetes.	Not including in sample because we lacked trial start and end dates.	374	Not included in sample because we lacked a trial start date.	6	61	N/A—supplement	SSED
Olympic Cool-Cap (2006)	Fitted cap that provides selective head cooling with mild systemic hypothermia to prevent or reduce the severity of neurologic injury in full-term infants with clinical evidence of moderate to severe brain injury due to lack of oxygen.	1523	936	2729	540	235	No	SSED; clinicaltrials.gov; FDA advisory committee documents
Cordis Precise Nitinol Stent System (2006)	Stent and delivery catheter system for high-risk patients with a neurological symptoms and stenosis of the common or internal carotid artery.	1826	1080	2243	360	747	Yes	SSED
Birmingham Hip Resurfacing System (2006)	Metal-on-metal resurfacing artificial hip replacement system to relieve hip pain and improve function by replacing parts of the hip that were damaged by degenerative joint diseases; intended for active patients who may not be suitable for traditional total hip replacement.	2496	659	3234	Not available	2385	Yes	SSED
Luma Cervical Imaging System (2006)	Optical detection system to help identify areas on the cervix with precancerous cells which may need to be biopsied; intended for use on women who recently had an abnormal Pap smear and are undergoing evaluation of the cervix.	420	636	1302	N/A—diagnostic	2526	No	SSED

## Results

We identified 27 approved device applications designated for priority review from January 2006 through August 2013. We excluded two products from some of our calculations due to a lack of complete information. These products were a pediatric glucose sensor and implantable miniature telescope because we lacked complete trial start and end times, and therefore could not calculate the length of the trial, time from the start of the trial until approval, or whether the application was submitted to FDA before the end of the trial (see Table 1).

The mean duration of pivotal trials was 3 years and 5 months, with a 3 year median. The briefest trial was only 3 months, for a continuous glucose monitor for diabetic patients. The longest pivotal trial lasted approximately 7 years, for a non-cemented ankle replacement that can simultaneously move in more than one direction (see Table 1).

The mean length of FDA review time was 1 year and 9 months, and the median time was 1 year and 3 months. This calculation, as previously mentioned, includes time when FDA is not reviewing the application as the agency awaits additional information from the product sponsor. The time from the initiation of the pivotal trial to FDA approval was a mean of 5 years and 1 month, with a median of 4 years and 8 months. The longest time period was more than 9 years, for a metal-on-metal hip resurfacing system. Of the 25 approved applications for which we have an end date for the pivotal study, 12 applications were submitted to FDA for review before the final extract date (see Table 1).

The mean and median duration of primary measure evaluation follow-up was 12 months, ranging from 6 days to nearly 3 years. We did not include five devices in this calculation that lacked patient follow-up; these included a computerized sedation system and several diagnostics, such as a device for detecting melanoma in atypical skin lesions (see Table 1).

Mean enrollment in pivotal trials was 663, with a median of 297. The smallest trial enrollment of 42 was for an endovascular stent graft used instead of open surgery to treat major arterial aneurysms. The largest enrollment was 2526 patients, for a diagnostic to identify precancerous cervical cells (see Table 1).

Among the 27 approved applications, three were already marketed in the United States for a different indication and four represented PMA supplements, which are submitted when manufacturers make changes affecting the safety or effectiveness of an approved product. Fifteen of the remaining 20 applications were available abroad, usually in Europe, at the time of FDA approval.

Additionally, FDA notified us that 11 devices—approximately 29 percent of submissions—with priority review status during this time period were ultimately rejected or withdrawn. Unapproved devices had a mean and median FDA review time of approximately 10 and 11 months, respectively [18].

## Discussion

There are limitations to this study. For example, the data included on clinical trials were not portrayed uniformly. Often, the terms used to describe the end dates of the trial differed, such as with descriptions of the date of the database lock, date of the final data extract or data collected on patients by a certain date. Additionally, the enrollment descriptions often referred to the number of patients, but could also reflect the number of procedures completed, such as with hip replacements. This variability often occurred because trial designs can differ substantially based on the product, and companies submit applications to FDA with varying narratives. We did note, however, that the more recent data sources were generally more complete and standardized. As previously referenced, we addressed the data variability in our calculations by supplementing the information with multiple data sources and omitting devices with incomplete data from our calculations. FDA and manufacturers should ensure that clinical summaries contain complete information to provide physicians, patients and researchers with the necessary data to evaluate the safety and effectiveness of new technologies.

Despite limitations, this study yields important data for clinicians and regulators on the clinical trials and review of priority review devices and suggests potential policy approaches.

First, roughly one-quarter of priority review applications were not ultimately approved; we found 27 approved applications and FDA identified 11 unapproved or withdrawn applications during our study time frame. This indicates that devices considered to be innovative advances were not able to meet FDA’s standards for safety and/or effectiveness—either because FDA notified the sponsor regarding data concerns or because the sponsor withdrew the application before obtaining an approval decision from the agency. Under FDA’s recent policy proposal—which would allow the approval of innovative devices based on smaller or shorter trials—the agency could have approved these products earlier for use in patient care based on less data than currently required. Under the EAP process envisioned by FDA, for some products, postmarket data may have then demonstrated that these devices did not meet the usual FDA standards of safety and effectiveness. This scenario is a distinct possibility, as some experts have pointed to examples of safety problems that have occurred with devices developed via other accelerated processes, such as the HDE program for rare disease products [19].

For this process to work—and to prevent clinicians from using unsafe or ineffective devices—FDA must have strict postmarket controls to ensure that sufficient data on the products are collected after approval. For devices where the postmarket data do not ultimately support FDA approval, the agency must also have sufficient authorities to quickly remove products from the market. Only through prompt data collection and product removal authorities can clinicians have assurances that devices reviewed via this new policy will improve patient outcomes.

Second, while FDA review times can last years, pivotal clinical trials account for more of the development time. As the speed of FDA review is often the focal point of discussions on facilitating device innovation, this finding highlights a potential target for new policies—including FDA’s new proposal—to accelerate the marketing of novel products by reducing the amount of time spent in clinical trials.

Third, the overall length of pivotal trials is substantially longer than the time needed for evaluating primary outcomes. This discrepancy is likely a result of difficulties in clinical trial enrollment—a challenge that could emanate from, among other things, patient disinterest, strict inclusion criteria, lack of incentives for patient or clinician participation, inadequate reimbursement when using exploratory products, Institutional Review Board approval challenges, and trial amendments that delay continuous enrollment [20–23]. The addition of secondary endpoints may also lengthen trials. FDA, manufacturers, researchers and other federal agencies—including the National Institutes of Health—should work together to develop solutions to reduce the discrepancy between primary outcome completion and overall trial length.

Last, the majority of devices were available abroad first. Without more information from Notified Bodies, it is impossible to similarly analyze the development and review of devices in Europe. However, our findings indicate that additional research and discussions are needed among the key stakeholders—including device manufacturers, clinical trial experts, FDA and academia—to identify appropriate reforms that do not put patients at undue risk but still enable prompt access to innovative devices in the United States. The EAP program, on which FDA obtained feedback from manufacturers, clinicians, patients and other stakeholders, can begin that conversation.

FDA’s new policy also has the potential to reduce the time that US patients and their physicians must wait for innovative treatment options, which are often already available abroad. Clinicians require these novel medical devices to save and improve patient lives, so long as the products are safe and effective.

Congress, FDA, manufacturers, physicians and other health policy stakeholders should work together to make sure that these and other new policies meaningfully shorten clinical development times to ensure that promising new technologies will reach US patients in a timely fashion and that clinicians have sufficient information to have confidence in the technologies used to treat, cure and diagnose serious medical conditions.
